# A Case of Thoracic Adhesions

**Published:** 1803-04-01

**Authors:** M. F. Wagstaffe

**Affiliations:** Southwark


### *A* Case *of* Thoracic Adhesions; *communicated by* Mr. M. F. Wagstaffe, *of Southwark.*

On the 16th of January, 1802, I was requested to attend the daughter of Mr. Morris, of the Borough, about fourteen years of age, thin spare habit, and sallow complexion, but of an active disposition, labouring under a severe attack of rheumatismus acutus, evidently characterised by

---

severe pain and swelling of the joints, glossy white skin, with considerable heat; tongue white, and some degree of dyspnœa. In this state, I gave her P. ipecac. c. in small doses, combined with magnesia and volatile tincture of guaiacum; other diaphoretics occasionally. The pulse was in general full, yet sometimes quick and weak, varying according to the quantity of rest, for which purpose opiates were occasionally resorted to. After attending her about ten days, and gaining but little advantage, a physician was consulted, who thought her in much danger; he prescribed ant. tart. gr. ß. calomelanos, gr. j. to be divided into four pills, taking one every six hours, with a diaphoretic draught, and an anodyne bolus at night; he found her much better on his third visit, and took his leave. This plan was continued a few days when the calomel was obliged to be omitted. She continued fluctuating for six weeks, when she left town, and returned in a short space of time, in apparent good health, continuing so till the beginning of October, when she was again attacked in the same manner. The disease had scarcely existed a week when the rheumatic symptoms disappeared, leaving a most distressing cough, excruciating pain in the left side, palpitation of the heart, and respiration much impeded, with great dread of suffocation; pulse from 100 to 140, sometimes throbbing, at other times weak. At this time I was induced to take five ounces of blood from the arm, which afforded temporary relief. The blood exhibited but slight marks of inflammation.

A few days after this, the respiration became very difficult, attended with occasional vomiting, and excruciating pain in the left side. In this state she was seen by another physician, who considered her situation extremely dangerous, from an idea of much adhesion about the heart and lungs, and the probability of effusion into the cavity of the chest. He directed tinct. digitalis from four to eight drops every six hours. emp. vesic. lateri affect. under which treatment she recovered very much, so indeed as to be able to eat with considerable appetite and retain her food on the stomach. She also had natural refreshing sleep. At this time she took small doses of calomel with digitalis; but after a few days she again relapsed into all the former distressing symptoms, and on November 23, expired in great agony.

It has been usual to consider acute rheumatism as a tedious rather than a dangerous disease. In the case before us, it becomes a question how far it was a remote cause of death death by inducing inflammation of the thoracic viscera? and it also excites inquiry whether that doctrine, (so generally propagated) which forbids the use of the lancet in acute rheumatism, is well founded?

Having permission to inspect the body, my friend Mr. Taunton kindly assisted me, when we found the appearances as follow:

On raising the sternum, the adhesion of the parts immediately underneath that bone was stronger than in a natural state, and compleatly obliterated the anterior mediastinum.

Both cavities of the thorax had suffered a similar fate, by the strong and extensive adhesions which had taken place.

On examining the left lung, which was placed in the posterior part near the spine, it was found very small, and adhering in every direction; the convex side to the pleura, lining the intercostal muscles, and the concave side to that part of the pleura which covers the pericardium. The substance of the lung appeared healthy.

The right lung adhered to every part with which it came in contact; its substance was natural.

The heart was enlarged, placed more to the left side, and the apex lower than natural.

Upon attempting to cut through the pericardium, it appeared of a firm consistence, and was so closely attached to the heart that it was very difficult in most parts, and in some quite impossible, to separate it.

The internal structure of the heart was healthy, and the valves natural.

The abdominal viscera were all healthy and in their usual situations.

I have taken the liberty to add three cases of peculiarity in the Cow-pox.

Case 1. I inoculated a child about three months old with genuine vaccine fluid, which excited inflammation and proceeded regularly till the 8th day, when the child was attacked with vomiting and diarrhœa, succeeded by convulsions, which terminated the next day in death.

Case 2. Another child, inoculated at the same time, proceeded regularly until the 9th day, when she was attacked with vomiting and diarrhœa, succeeded by convulsions, which continued six weeks, and then terminated fatally.

I shall make no comments on these cases, as I could much wish for the sentiments of some of your correspondents who have seen more of the disease than myself.

The first child was in perfect health when inoculated; the second had a troublesome cough.

Case 3. A few weeks ago I inoculated a child with vaccine fluid in both arms, one of which inflamed, and went through the usual process; the other, as I supposed, had failed. On the 13th day after inoculation the child was taken into the country, and in the course of two or three days the nurse observed the other arm inflamed; the inflammation continued to spread, and maturated at the usual time; the only difference between the two was, that the latter pustule was not so large as the former.

*Southwark, Feb.* 18, 1803.

